# A new record of the occurrence of *Trichuris skrjabini* Baskakov, 1924 in goats of Pakistan

**DOI:** 10.1371/journal.pone.0290906

**Published:** 2023-09-01

**Authors:** Kiran Afshan, Sabahat Khan, Bilal Khan, Sobia Hussain, Sabika Firasat, Ghulam Narjis, Umer Chaudhry

**Affiliations:** 1 Faculty of Biological Sciences, Department of Zoology, Quaid-i-Azam University, Islamabad, Islambad, Pakistan; 2 Department of Statistics, Rawalpindi Women University, Satellite Town, Rawalpindi, Punjab, Pakistan; 3 School of Veterinary Medicine, St. George’s University True Blue, Grenada, Caribbean, West Indies; Universidade Federal de Minas Gerais, BRAZIL

## Abstract

More than 23 Trichuroidea species have been identified in ruminants in different parts of the world. Most are pathogenic, causing trichurosis. *Trichuris* adults of most species within this family have a predilection for the ceca, where they may cause damage to the epithelial wall. In the present study, *Trichuris* spp. from large intestine of goats were analysed based on morphological and molecular characteristics. Fifty adult worms (male = 25 and female = 25) were selected for morphometric and molecular analysis. Male *Trichuris* were distinguished by their longer spicules, typical spicule sheaths, and small spicules that were always completely covered by the spicule sheath. The presence of an uneverted vulva in the vagina distinguished female worms. We have performed the molecular characterisation of adult warms to identify as *Trichuris skrjabini*. Genetic comparison of *T*. *skrjabini* rDNA ITS2 sequences with those from other *Trichuris* spp. was performed to assess within and between species variation and validate the use of ITS-2 rDNA as a robust species-specific marker for *T*. *skrjabini* identification. This work provides the first report of this parasite species from Pakistan and validated species-specific marker of *T*. *skrjabini* that reduces the production potential of goats in the country.

## Introduction

*Trichuris* (Roederer, 1761 –Nematoda: Trichuridae) infect a wide variety of mammalian hosts, including humans, ruminants, marsupials, rodents, and primates [1]. More than 23 species of *Trichuris* have been identified in ruminants [2], with the most common being *Trichuris ovis*, *Trichuris skrjabini*, and *Trichuris discolor* [3,4].

Different biometric and morphological traits have been employed in the taxonomic characterisation of the *Trichuris* species [5]. Only a few characteristics have been described, and congeneric species have not been sufficiently compared [6]. Although morphometric analysis provides keys for *Trichuris* identification, it is a more traditional approach that might lead to ambiguity due to overlapping morphological and morphometrical traits leading to taxonomic and nomenclatorial challenges [7].

In taxonomic groups of *Trichuris* with complex systematics and overlapping characterised by morphological convergence, using molecular methods to identify *Trichuris* at the species level is essential [8,9]. Numerous helminth investigations have used the ITS-2 of the ribosomal DNA (rDNA) as their target locus. This region is considered an acceptable genetic marker for resolving links at the species level [10]. Molecular methods have shown that not all species initially described by morphometric characteristics would remain fully identified [11,12]. Therefore, it is essential to use rDNA regions encompassing the ITS2 to identify *Trichuris* from sympatric areas worldwide [10,13]. Pakistan has an agriculture-based economy, with livestock being an integral part. The distribution of *Trichuris* in goats has been sporadically investigated in Pakistan. A few studies previously described *Trichuris ovis* through egg and adult morphology isolated from sheep and goats, whereas most simply identified at the genus level [14–19]. *Trichuris suis* (pig whipworm) and *Trichuris vulpis* (canine whipworm) are considered zoonotic parasites which can threaten the human population [20] and require more reliable methodologies to improve the knowledge of the *Trichuris* species. However, much of the discussion around integrative taxonomy deals with the merits of applying morphological versus molecular characteristics [21–23]. The present study is the first to confirm the species identity of *Trichuris skrjabini* from goats using the rDNA ITS-2 genetic marker. Our results suggest, in contrast to previous morphologically based studies, that *Trichuris skrjabini* is the predominant goats species in the Punjab province, Pakistan. There is no evidence of *Trichuris ovis* found in this study.

## Materials and methods

### Ethical approval

The collection of worms from the slaughtered animals does not require ethical approval, the animals were slaughtered for other purposes to meet the high protein demand of population. The study was approved by the Animal Ethics Committee of the Quaid-i-Azam University,Islamabad.

### Worm collection

In this cross-sectional study, 231 slaughtered goats were examined between February and August 2022 at the Sihala abattoir in the Rawalpindi division of the Punjab province. The sample size was determined by using the formula [24],

$$n=\frac{Z^{2}P(1-P)}{d^{2}}$$ (1)

where n was the sample size, Z was the desired confidence interval (95%), P was the expected proportion of infected animals in the population (0.35) and d was precision of estimation (5%). The calculated sample size was 188, but we increased it to 231 for greater precision. The animals were examined using a convenient random sample method. Fifty worms (2 to 3 worms per host) were recovered from the cecum or the cecal epithelial wall. The worms were rinsed in phosphate-buffered saline (PBS) before being preserved in 70% ethanol for morphometric and molecular analysis.

### Morphological examination

50 adult warms (male = 25 and female = 25) were fixed for morphometric analysis. The worms were cleared in lactophenol and identified under the microscope [25]. Standardised measurements were obtained among the male characters, and spicule length is regarded as the most important criterion for differentiating *Trichuris* [26]. The main factors used to identify females are the vulvar morphology [27], the structure and lining of the vagina and, alternatively, the distance from the vulva to the uterine sphincter [3,8]. The descriptive univariate statistics based on mean values, standard deviation and range for all parameters were determined for male and female worms [9].

### Genomic DNA extraction

Genomic DNA was extracted from 15 out of 50 individual worms of either sex. A small tissue sample of 2 mg was taken from each worm head and put in a petri dish with distilled water (dH2O) to prevent egg contamination. Each worm piece was rinsed twice for 5 min to remove all traces of ethanol. The worms were lysed in a 25 μl worm lysis solution made by mixing 50 μl of proteinase K (10 mg/ml, New England Bio Labs) in 1 ml of Direct PCR lysis reagent (Viagen) following the lysates were incubated for 2 hrs at 60°C and then for 15 min at 85°C [28].

### PCR amplification of ITS-2 ribosomal DNA

A total of 500 bp of the ITS-2 region of the rDNA was amplified by using previously reported *Trichuris*-specific forward primer (ITS2-F: CTCGTAGGTCGTTGAAGAAC) [29] together with universal reverse primer (ITS2-R: TTAGTTTCTTTTCCTCCGCT) [30]. The 25 μl PCR reaction mixtures consisted of 2 μl of PCR buffer (1×) (Thermo Fisher Scientific, USA), 2 μl MgCl_2_ (25 mM), 2 μl of 2.5 mM dNTPs, 0.7 μl of the primer mix (10 pmol/μl final concentration of each primer), 2 μl of gDNA, and 0.3 μl of Taq DNA polymerase (5 U/μl) (Thermo Fisher Scientific, USA) and 16 μl ddH20. The thermocycler conditions were set at 96°C for 45 sec, followed by 35 cycles of 95°C for 45 sec, 50°C for 45 sec, and 72°C for 45 sec, with a final extension procedure of 72°C for 10 min. The amplified products were visualised by electrophoresis in 1% agarose gel (S1 Fig) PCR products were cleaned using a WizPrepTM Gel/PCR Purification Mini kit (Seongnam 13,209; South Korea).

### Sequence and phylogenetic analysis of ITS-2 ribosomal DNA

PCR products were submitted for commercial sequencing (Macrogen, Korea), using the same amplification primers. Both strands of rDNA ITS-2 sequences from each worm were assembled, aligned, and edited to remove primers from both ends using the MUSCLE Alignment tool of the Geneious Pro 5.4 software [31]. The CD-HIT Suite software grouped sequences showing 100% base pair similarity into single unique sequence variants [32]. The unique sequence variants were further aligned with previously published NCBI GenBank rDNA ITS-2 of *Trichuris* species. All sequences of field samples and the GeneBank were trimmed to 388 bp, the length of the shortest sequence available that contained all the informative sites. The phylogenetic analysis was inferred using the Maximum Likelihood method and Kimura 2-parameter model [33]. The tree with the highest log likelihood (-2414.08) is shown. The percentage of trees where the associated taxa clustered together is displayed next to the branches. Initial tree(s) for the heuristic search were obtained automatically by applying Neighbor-Join and BioNJ algorithms to a matrix of pairwise distances estimated using the Maximum Composite Likelihood (MCL) approach and then selecting the topology with superior log likelihood value. A discrete Gamma distribution was used to model evolutionary rate differences among sites [5 categories (+*G*, parameter = 7.5222)]. The tree is drawn to scale, with branch lengths measured in the number of substitutions per site. All positions containing gaps and missing data were eliminated (complete deletion option).

## Results

### Morphological description

Family Trichuroidea (Ransom, 1911) Railliet, 1915

Genus *Trichuris* Roederer, 1761

*Trichuris skrjabini* (Baskakov, 1924)

### General characteristics of *Trichuris*

**Fig 1:**
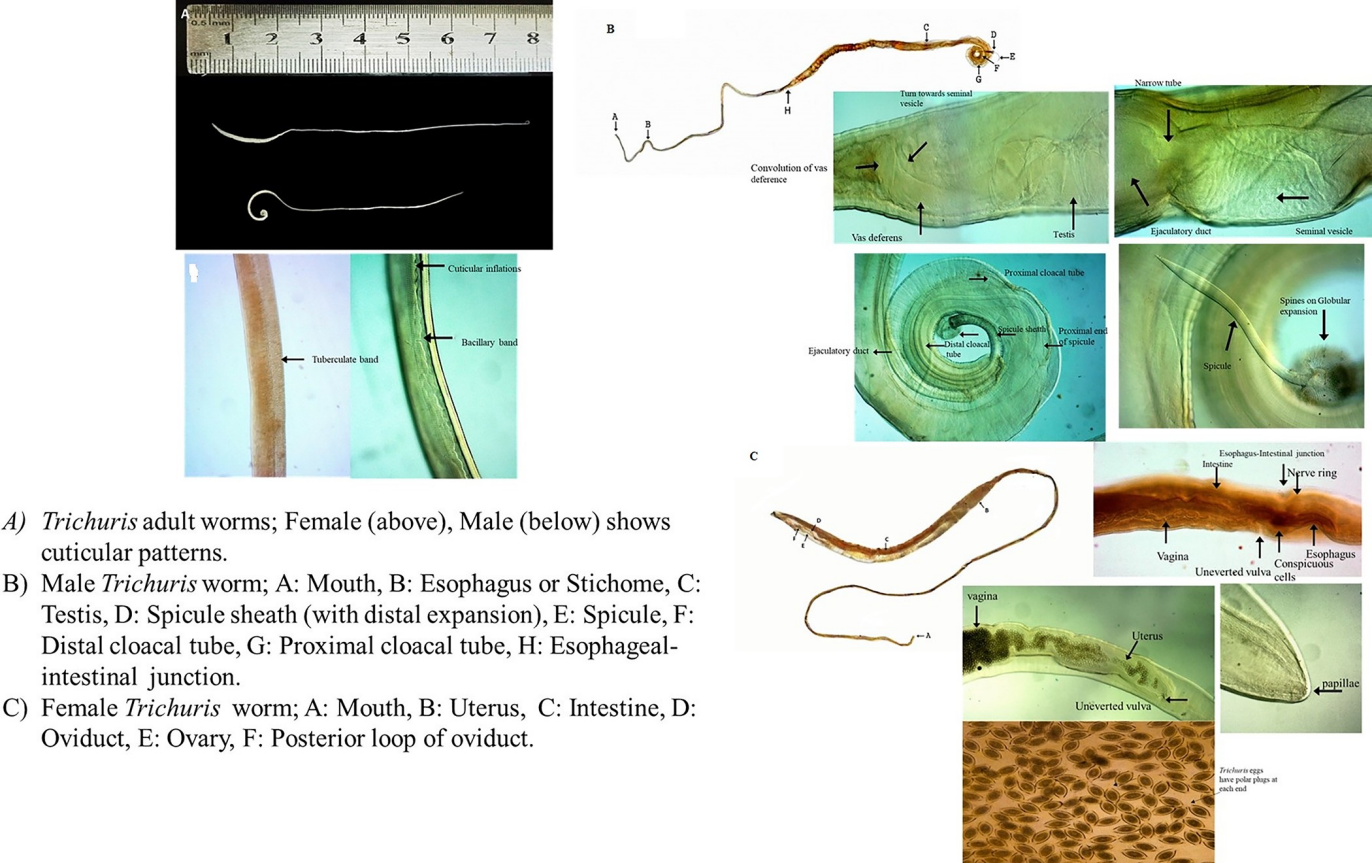
A light micrograph of *Trichuris* from the present study.

Morphological characters revealed that the worms had a filiform anterior half and a broad handle-like posterior part (Fig 1). The narrow anterior part displays two different cuticular patterns. One side is heavily striated with transverse grooves, while the other is a delicately tuberculate band showing small circular elevated bodies uniformly spaced (Fig 1A). The anterior part of the body has a ventral side that tapers somewhat toward the cephalic end, revealing a broad longitudinally elongated "bacillary band" with typical cuticle inflations. A set of cephalic papillae surrounds the mouth, formed in two circles (an inner circle and a lateral circle), with a prominent organ, the stylet, emerging from the mouth cavity’s central portion.

### Male worms characteristics

The body is 25–49 mm (36.6±7.65) long. The ratio between anterior and posterior body length is 2:1. The thin anterior part is 0.66:1 of the entire body length. The length of the oesophagus is 18–38 (24.4±7.08). The body’s posterior end is curved ventrally. The width at oesophageal region of body is 0.11–0.18 (0.16±0.02), the width at the level of oesophagus-intestinal junction is 0.21–0.75 (0.45±0.19), and the width of the posterior region of body is 0.57–0.96 (0.7±0.14). The distance from the head to the beginning of the bacillary stripes is 0.8–0.98 (0.86±0.06), and the length of the bacillary strips is 1.8–2 (1.92±0.07).

The reproductive system has a spicular tube with a length of 4.31–5.29 (5.0±0.43), diameter of spicule is 0.012–0.017 (0.014± 0.001), and the width of the proximal end of the spicule is 0.02–0.04(0.027±0.007). The maximum length of the spicular sheath is 1.07–1.4 (1.21±0.11), the width of the spicular sheath is 0.25–0.4(0.29±0.05), and the width of the spicular sheath at the tail end of body is 0.25–0.4 (0.29±0.05). The distal bulb/expansion length measurement is 0.13–0.21 (0.16±0.02), and the distal bulb/expansion width is 0.28–0.43(0.336±0.05). The testis is the first part of the genital apparatus, and it is very long and highly convoluted, starting in the posterior part of the male body, oriented anteriorly, and extending along the longitudinal axis of the body, finishing not far from the oesophagus transition into the intestine. The vas deferens run somewhat anteriorly along the intestine, connect the testis in the initial component of the genital apparatus and are pretty lengthy and convoluted to the ejaculatory duct via a tiny tube. The distance between posterior part of testis and tail end of body is 2.85–10.4(6.44±2.75). The length of the ejaculatory duct is 2.57–4.8(4.23±0.86), length of the cloaca is 3.51±0.48 (3.2–4.45). The spicule is in the distal cloacal tube, and protrudes from the sheath in a distinctive spherical expansion. The spines on distal expansion are longer than those on the spicular sheath (Fig 1B).

### Female worms characteristics

The body is 36–69 mm (50.4±12.46) long. The ratio between anterior and posterior body length is 1.75:1. The thin anterior part is 0.63:1 on the entire body length (Fig 1C). The total length of the oesophagus is 18–52 (32±12.37). The width of the oesophageal region of the body is 0.15–0.22 (0.19±0.02), the body width at the level of the oesophagus-intestinal junction is 0.31–0.87 (0.47±0.20), the width of the posterior region of body is 0.7–1.1 (0.81±0.14). The distance from the head to the beginning of the bacillary stripes is 0.96–1.37 (1.26±0.15), and the length of bacillary stripes is 1.3–2.3 (1.68±0.37).

A single uterus and vulva are non-protrusive and with a length of 0.02–0.49 (0.24±0.15) at the confluence of the oesophagus and the intestine. The length of muscular zone of the oesophagus is 0.71–2 (1.00±0.49). The vagina is lengthy, about 2.3–4.45 (0.24±0.15), with circumvolutions close to the uterus, a brief straight zone, an extended middle part, and one gently coiled straight portion at the vulva. The distance of the uterus’s posterior loop from the body’s tail end is 0.14–1.24(0.92±0.41). The ovary is lengthy and connects to the oviduct in the back of the body. The distance of tail end of body and posterior fold of seminal receptacle is 0.34–0.5 (0.41±0.05). At the end of the tail, the anus is sub-terminally located (Fig 1C). The eggs found in the gravid uterus were barrel-shaped and had clear mucoid-appearing polar plugs with a brown outer covering (Fig 1C).

Taxonomic summary

Type host: Goat (*Capra aegagrus hircus*)

Site of infection: Cecum

Type locality: Rawalpindi Punjab

### Genetic analysis of *Trichuris*

500 bp rDNA ITS-2 fragments were PCR amplified and sequenced from the abattoir-derived *Trichuris* collected from goats. All sequences in the alignment were trimmed to 388 bp (S2 Fig). The chromatogram revealed that 11 of the 15 different worms had clean rDNA ITS-2 sequences (https://data.mendeley.com/datasets/2mv8ngsg6p/1). Those 11 *Trichuris* sequences were aligned with 14 NCBI GeneBank sequences of different *Trichuris* species (3 sequences from *Trichuris spp*., 2 sequences from *Trichuris skrjabini*, one sequence from *Trichuris leporis*, 3 sequences from *Trichuris ovis*, one sequence from *Trichuris colobae*, 2 sequences from *Trichuris trichiura*, 2 sequences from *Trichuris suis*).

**Fig 2:**
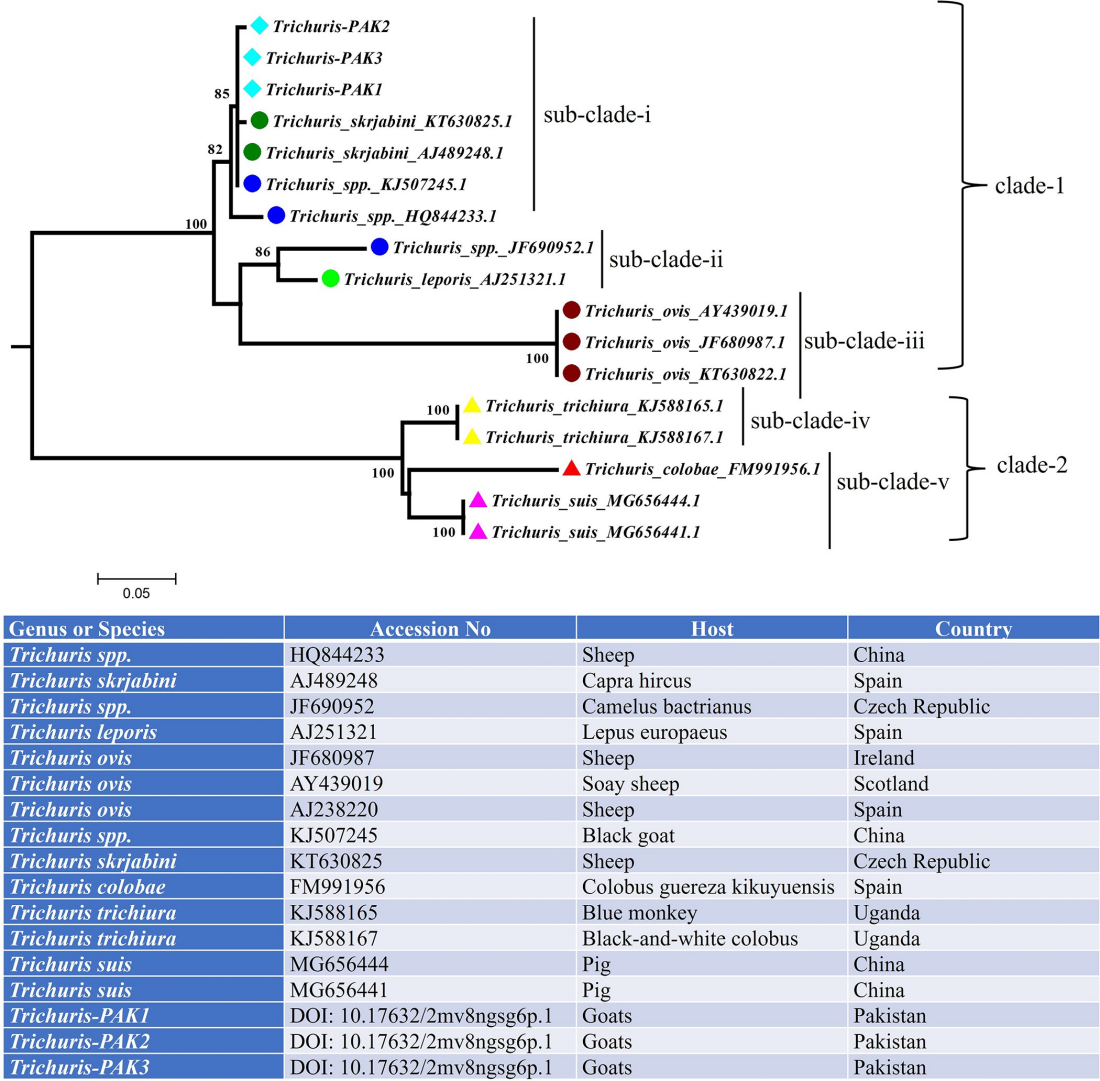
Maximum-likelihood tree was obtained from the rDNA ITS2 sequences of *Trichuris*. 11 Three unique sequence variants of *Trichuris* were aligned with 14 NCBI GeneBank sequences of different *Trichuris* species. The sequences of each species were identified with the name and color circles. The adjacent table indicates the accession number, host, and country of origin.

The genetic distance search of 11 *Trichuris* rDNA ITS2 sequences (present study) shows 99–100% similarity to a previously identified *Trichuris skrjabini* (AJ489248, KT630825), *Trichuris* spp. (KJ507245) and 96% similarity with *Trichuris* spp. (HQ844233) (Table 1). The further comparison showed the lowest similarities (42–78%) between *Trichuris* of the present study and previously identified rDNA ITS2 sequences of *Trichuris spp*. (HQ844233, JF690952) *Trichuris leporis* (AJ251321) *Trichuris ovis* (JF680987, AY439019, AJ238220), *Trichuris suis* (MG656444, MG656441), *Trichuris trichiura* (KJ588165, KJ588167) and *Trichuris colobae* (FM991956) (Table 1, Fig 2). The full sequence analysis of 388 bp of the rDNA ITS-2 locus revealed four intraspecific variations (P66, P74, P114, P178) in the 11 *Trichuris* sequences generated by the present study and *Trichuris skrjabini* (AJ489248, KT630825), *Trichuris* spp. (KJ507245) indicate genetically linked to the rDNA ITS2 sequence.

**Table 1:** Estimates of evolutionary divergence between sequences of *Trichuris* species. This analysis involved 25 nucleotide sequences. The percentage of base substitutions per site between sequences is shown. The analyses were conducted using the Maximum Composite Likelihood model. All positions containing gaps and missing data were eliminated. There was a total of 318 nucleotide positions in the final dataset.

	Trichuris spp.	Trichuris spp.	Trichuris ovis	Trichuris ovis	Trichuris ovis	Trichuris leporis	Trichuris colobae	Trichuris trichiura	Trichuris trichiura	Trichuris suis	Trichuris suis	Trichuris skrjabini	Trichuris skrjabini	Trichuris spp.
	HQ844233.1	JF690952.1	AY439019.1	JF680987.1	KT630822.1	AJ251321.1	FM991956.1	KJ588165.1	KJ588167.1	MG656444.1	MG656441.1	KT630825.1	AJ489248.1	KJ507245.1
Trichuris spp. JF690952.1	72													
Trichuris ovis AY439019.1	56	58												
Trichuris ovis JF680987.1	57	59	99											
Trichuris ovis KT630822.1	57	59	99	100										
Trichuris leporis AJ251321.1	77	77	58	59	59									
Trichuris colobae FM991956.1	41	38	39	39	39	41								
Trichuris trichiura KJ588165.1	44	39	40	40	40	44	71							
Trichuris trichiura KJ588167.1	43	38	37	37	37	44	63	91						
Trichuris suis MG656444.1	44	39	40	40	40	44	78	75	71					
Trichuris suis MG656441.1	44	39	40	40	40	43	78	74	70	98				
Trichuris skrjabini KT630825.1	96	73	58	58	58	77	42	45	44	45	45			
Trichuris skrjabini AJ489248.1	96	73	58	58	58	78	42	45	44	45	45	100		
Trichuris spp. KJ507245.1	96	73	58	58	58	78	42	45	44	45	45	99	100	
Trichuris-1 PAK	96	73	58	58	58	78	42	45	44	45	45	99	100	100
Trichuris-2 PAK	96	73	58	58	58	78	42	45	44	45	45	99	100	100
Trichuris-3 PAK	96	73	58	58	58	78	42	45	44	45	45	99	100	100
Trichuris-5 PAK	96	73	58	58	58	78	41	45	44	45	45	99	99	100
Trichuris-6 PAK	96	73	58	58	58	78	42	45	44	45	45	99	100	100
Trichuris-7 PAK	96	73	58	58	58	78	42	45	44	45	45	99	100	100
Trichuris-8 PAK	96	73	58	58	58	78	42	45	44	45	45	99	100	100
Trichuris-11 PAK	96	73	58	58	58	78	42	45	44	45	45	99	100	100
Trichuris-12 PAK	96	73	58	58	58	78	42	45	44	45	45	99	100	100
Trichuris-14 PAK	97	73	58	58	58	78	42	45	44	45	45	99	99	100
Trichuris-15 PAK	96	73	58	58	58	78	42	45	44	45	45	99	100	100

Eleven *Trichuris* sequences were further grouped into three unique sequence variants (*Trichuris-PAK1*, *Trichuris-PAK2*, *Trichuris-PAK3*) and aligned with 14 NCBI GeneBank sequences of different *Trichuris* species. The phylogenetic tree was generated intwo separate clades. In clade 1, three unique sequence variants from Pakistan revealed a close link with *Trichuris skrjabini* (AJ489248, KT630825) and *Trichuris* spp. (KJ507245, HQ844233) within subclade-i (Fig 2). The other two subclades (ii and iii) include *Trichuris* spp. (JF690952), *T*. *leporis* (AJ251321) and *T*. *ovis* (JF680987, AY439019, AJ238220) species. Similarly, the phylogenetic comparison showed that *T*. *suis*, *T*. *colobae*, and *T*. *trichuris* form two distinct subclades (iv and v) within clade 2 (Fig 2).

## Discussion

It is assumed that *T*. *ovis* were the predominant species in the Punjab province of Pakistan [14–19]. However, all these reports were based on egg and or adult morphology without genetic confirmation of species identity. In the present study, adult *Trichuris* infecting goats were characterised by the adult morphology at the genus level and the sequences of rDNA ITS-2 region, providing the first documented report of *T*. *skrjabini* in the Rawalpindi division of the Punjab province.

First, we have performed the morphological characteristics of *Trichuris* genus infecting goats. The male of the genus *Trichuris* displayed a similar morphological pattern in the reproductive system. Additionally, the characteristic of the spicule sheath, spicular length, and the fact that the spicule sheath covers a short spicule was identified in male [34]. The presence of vagina with a noneverted vulva allows for distinguishing between the females [35]. *Trichuris skrjabini* morphological characters were compared with *T*. *ovis* [Baylis (1932), Ortlepp (1937) and Sarwar (1937)]. Morphological characters used for the routine separation of *T*. *ovis* from *T*. *skrjabin* without the necessity for measurement in the male are the size, shape and degree of eversion of the spicule, and in the female, the type of vulval expansion, nature of the vagina and straight portion of the ovary [36–38]. However, the length and breadth of egg range overlap between the two species. All the measurements of *T*. *skrjabini* recorded in the present study are within the range described by Baskakov [39], Magomedbekov [40], Knight [36] and Hinks and Thomas [38]. The speciation of the *Trichuris* genus is challenging because of the *Trichuris* phenotypic plasticity, lack of morphological features, and the substantial overlap in morphometric traits among species [41].

Therefore, 11 *Trichuris* rDNA ITS-2 sequences from the present study and 14 *Trichuris* sequences from NCBI GenBank were examined. The comparison between *Trichuris* of the present study yielded 99–100% similarity to a previously identified rDNA ITS2 sequence of *Trichuris skrjabini* (KT630825, AJ489248) isolated from the sheep in Czech Republic and goats in Spain and *Trichuris* spp. (KJ507245) isolated from the black goat in China. Similarly, phylogenetic analysis indicates that *Trichuris* from Pakistan revealed a strong genetic link between *T*. *skrjabini* descended from sheep in Czech Republic and goats in Spain and *Trichuris* spp. descended from goats in China. We have identified four intraspecific single nucleotide polymorphisms (SNPs) between sequences of the *Trichuris* generated by the present study and *Trichuris skrjabini* (AJ489248, KT630825), *Trichuris* spp. (KJ507245).

## Conclusions

In conclusion, the molecular confirmation and the phylogenetic analysis of the intestinal nematode of goats confirm, for the first time, the presence of *Trichuris skrjabini* in the Rawalpindi division of Punjab. Furthermore, *Trichuris skrjabini* was the only nematode identified in 11 worms from goats. The results of our study have implications for the diagnosis and control of *Trichuris* in the region and the need for accurate species identification to understand parasite distribution and population genetics. There is a need to identify new genetic markers for molecular analysis of a wide range of *Trichuris* isolates from different host species and geographical areas to improve our understanding of parasite population genetic structures and transmission dynamics.

## Supporting information

S1 Fig Agarose gel electrophoresis (1%) of PCR product band of 500 bp of *Trichuris* the ITS-2 region. (PNG)

S2 Fig Sequences alignment trimmed to 388 bp. (JPG)
