# Correction: A new record of the occurrence of *Trichuris skrjabini* Baskakov, 1924 in goats of Pakistan

**DOI:** 10.1371/journal.pone.0319514

**Published:** 2025-02-12

**Authors:** The PLOS ONE Staff

The authors, Kiran Afshan and Umer Chaudhry, should be noted as co-corresponding authors of this work. Kiran Afshan email address is: kafshan@qau.edu.pk.

The publisher apologizes for the error.
