# Supplementary figures and images for: A new record of the occurrence of Trichuris skrjabini Baskakov, 1924 in goats of Pakistan
Source: PLoS One. 2023 Sep 1;18(9):e0290906. doi: 10.1371/journal.pone.0290906 (PMC10473508; doi:10.1371/journal.pone.0290906)

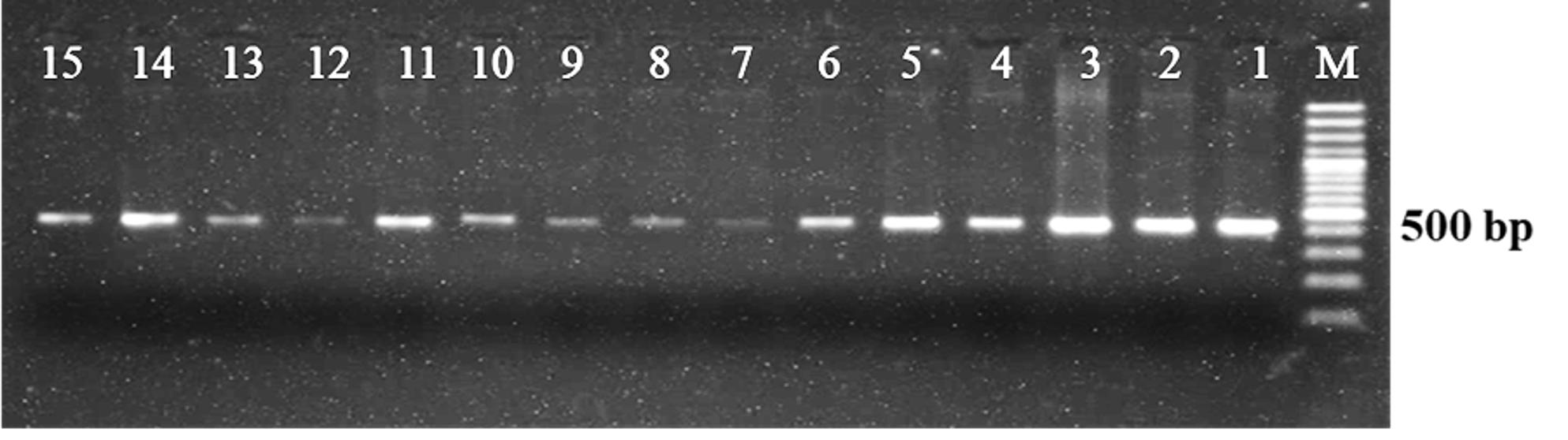

Supplement: S1 Fig — (PNG) [file pone.0290906.s001.png]

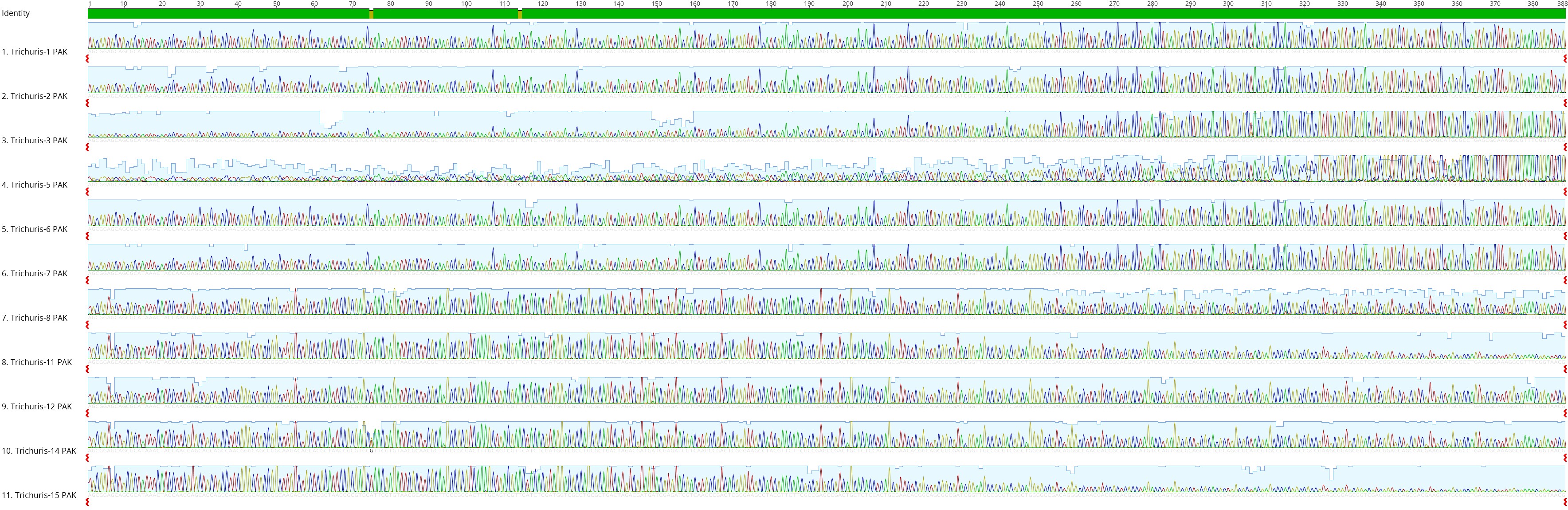

Supplement: S2 Fig — (JPG) [file pone.0290906.s002.jpg]
